# Can China afford rapid aging?

**DOI:** 10.1186/s40064-016-2778-0

**Published:** 2016-07-18

**Authors:** Quanbao Jiang, Shucai Yang, Jesús J. Sánchez-Barricarte

**Affiliations:** Institute for Population and Development Studies, Xi’an Jiaotong University, Xi’an, China; Carlos III University of Madrid, Madrid, Spain

**Keywords:** Aging, Health status, Social security system, Pension, Long-term care, China

## Abstract

China’s rapid aging has caused widespread concern, but it seems that the situations and consequences of rapid aging are not adequately acknowledged. This study analyzed the problem of ageing in China from the aspects of elderly people’s health status, income source, daily care, suicide, the weak social security system in terms of pension, health expenses, and long-term care costs as well as incoming accelerating ageing process in China. All these factors indicate that it is difficult for China to afford the issue of a rapidly aging population.

## Background

With the sharp decline in China’s fertility rate (from the census data of 2.63 in 1982 to 1.18 in 2010) and the great rise in life expectancy (from 67.77 years old in 1981 to 74.83 in 2010), both the number and proportion of the elderly in China have risen rapidly. In 1982, there were 49.28 million people aged 65 and over, accounting for 4.91 %. In 2000, the number of those aged 65 and over rose to 88.27 million (7.10 %). By 2010, the number of the elderly aged 65 and over had reached 118.93 million (8.92 %). In rural areas, the population aged 65 and over was 66.67 million, which accounted for 10.06 % (PCO 2002, 2012).

The Chinese government has also realized that China’s rapid aging is an issue. In the Decision of the Central Committee of the Communist Party of China on Some Major Issues Concerning Comprehensively Deepening the Reform passed at the Third Plenary Session of the 18th Central Committee of the Communist Party of China held in November 2013, the government stated that they would respond actively to the aging of the population, quicken steps to establish a social endowment service system and develop the service industry for the elderly, and improve the system of care for seniors. However, this resolution includes no detailed measures to actively respond to the aging of the population.

With the development of China’s population, society and economy, Chinese family size is shrinking and the number of children is dwindling; urbanization brings numerous young people to cities, but the social security system is not well established yet as far as pensions are concerned. All these aging-related issues pose considerable challenges (CHARLS Research Team 2013; Zhao et al. 2014).

**Fig. 1:**
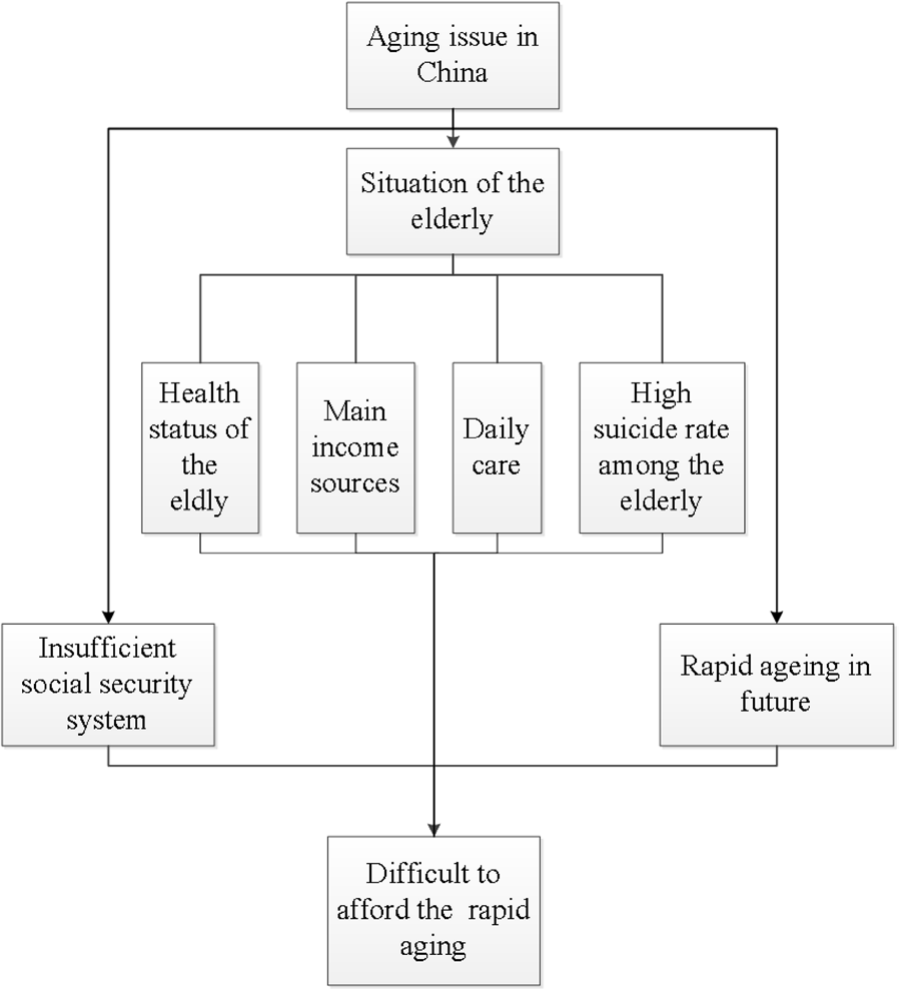
The analytical framework

As indicated in Fig. 1, first of all, we analyze the life circumstances of the elderly in terms of their health status, main income sources and the daily care they receive, and describe the current situation, in which the elderly may even commit suicide due to impoverishment. Secondly, we analyze this situation and the limitations of China’s social security system, including the pension system, and the cost of healthcare and long-term care. Finally, we examine the future trends regarding China’s aging population through the predicted data. Combining all these three main aspects, we discuss whether China can deal with its aging population. It is expected that this analysis will draw close attention from the Chinese government, alert the government to the issue of aging, and accordingly lead to the formulation of practical, feasible measures for responding to the aging population.

## The situation of the elderly

### Health status of the elderly

The data from China’s 2010 population census divided the health status of the elderly into four kinds, i.e., healthy, basically healthy, unhealthy but able to take care of oneself, and not able to take care of oneself. The data indicated that in the elderly population aged 65 and over, healthy people accounted for 35 %, basically healthy people, 43 %, those not healthy but able to take care of themselves, 18 %, and those unable to take care of themselves, 5 %. In the population aged 80 and over, the percentage of these four population groups is 19, 41, 29 and 10 % respectively. In the rural elderly aged 65 and over, the percentage of the four population groups, i.e., healthy, basically healthy, unhealthy but able to take care of oneself, and not able to take care of oneself, is 32, 42, 22 and 4 % respectively. In the rural elderly aged 80 and over, the percentage of the four groups is 16, 39, 34 and 11 % respectively (PCO 2012). Moreover, microcosmic survey data indicated that health status of the elderly is even worse than this seems to indicate. In a self-rated health survey, 31.8 % of elderly people stated that they were in bad or worse health, 38.1 % had physical disabilities (having difficulty in completing basic daily activities), 23.8 % reported needing help in basic daily activities, and 33.4 % reported physical pain (CHARLS Research Team 2013).

China’s elderly people have major difficulties with the Instrumental Activity of Daily Living (IADL) or Activities of Daily Living (ADL) (For IADL disability and ADL disability, see Gu et al. 2010). Based on the data from the Chinese longitudinal healthy longevity survey (CLHLS) 2008 and 2011 carried out by Peking University, using the multi-state life table method, we calculated the life expectancy of people aged 65 as 16.3 years, including 8.7 years of healthy status, 4.8 years with only difficulty in IADL, and 2.8 years with difficulty in ADL. In view of the different health status of the people aged 65, the years of each kind of status for life expectancy vary, as shown in Table 1. For the people aged 80, the life expectancy is 7.4 years, including 2.0 years of healthy status, 3.4 years with only difficulty in IADL, and 2.0 years with difficulty in ADL. In view of the different health status of the people aged 80, the years of each kind of status for life expectancy vary, as listed in Table 1.

**Table 1:** Remaining life expectancies by health status (years)

Remaining life expectancy by status	Status at 65				Status at 80			
	All	Disability-free	Only IADL disabled	ADL disabled	All	Disability-free	Only IADL disabled	ADL disabled
Disability-free	8.7	9.1	4.4	2.1	2.0	3.7	0.7	0.2
Only IADL disabled	4.8	4.6	8.7	3.0	3.4	2.4	4.8	1.0
ADL disabled	2.8	2.7	3.1	9.9	2.0	1.6	1.7	5.8
Total	16.3	16.3	16.2	14.9	7.4	7.7	7.2	7.0

*Data Source* Calculated using CLHLS data

Health status can affect the elderly in many ways. Above all, if the elderly are not well enough to work, they can barely earn an income; thus, older people in rural areas may rely heavily on the support of their family members. Next come living arrangements. Since the disabled elderly need long-term care, they often need to live with their adult children. Finally, in terms of the healthcare they receive, spending on health care and family care for ill elderly people is on the rise (Du 2013).

### Main income sources

The data from China’s 2010 population census indicated that as far as the main sources of support for people aged 65 and over are concerned, 49 % comes from the support of other family members, 20 % comes from their labor income and 25 % from pensions for the elderly and retired veterans. As they get older, the elderly gradually lose labor capacity and rely more and more on the support of other family members. For people aged 65, 40 % take labor income as main source of support; for 70-year-olds, the percentage drops to 23 %; for people aged 80, it is 5 %. At the same time, 31 % of people aged 65 have the main source of support coming from other family members; for the people aged 70, the percentage is 44 %, and for the people aged 80, the percentage rises to 63 % (PCO 2012).

Regarding the dual urban–rural structure in China, there are enormous differences between urban and rural areas in methods of elderly care. In urban areas, the main source of support for people aged 65 and over comes first from the pensions for the elderly and retired veterans, accounting for 67 %; it comes secondly from the support of other family members, accounting for 24 %; labor income only takes up 4 %. In rural areas, the main source of support for people aged 65 and over comes first from the support of other family members, accounting for 59 %; 28 % rely on their labor income; only 5 % rely on pensions for the elderly and retired veterans (PCO 2012). Elderly women rely more on the support of other family members. In the whole of China, 60 % of women aged 65 and over rely mainly on the support of other family members; in rural areas, the percentage is even as high as 71 % (PCO 2012).

Table 2 shows that the elderly’s main income sources vary with their health status. For people aged 60 and over, as their health gets worse, they become less dependent on their own labor income as their main source of support. The proportion of people taking their labor income as their main source of support decreases from 41.95 % for healthy seniors to 1.16 % for those disabled ones, while the proportion of people depending mainly on the support of other family members as their main income source rises from 26.49 % for healthy seniors to 70.33 % for those who cannot take care of themselves. The poorer health the elderly are in, the more dependent they are on the support of other family members.

**Table 2:** Distribution of income sources for people aged 60 and over by health status (%)

Income sources	All	Healthy	Basically healthy	Unhealthy but able to take care of oneself	Not able to take care of oneself
Support of other family members	40.72	26.49	44.67	68.12	70.33
Labor income	29.07	41.95	24.75	6.64	1.16
Pensions for the elderly and retired veterans	24.12	28.00	24.45	12.58	16.32
Income from transfers	0.37	0.42	0.36	0.27	0.20
Guaranteeing the lowest living	3.89	1.69	3.8	9.84	9.88
Others	1.83	1.46	1.97	2.55	2.11
All	100	100	100	100	100

*Data source* PCO (2012)

One important point is that a small proportion of the elderly receive pensions for the elderly and retired veterans as their main income source, and most of these people live in urban areas. The social security systems for the elderly who live in rural areas are at a low level. On the other hand, the percentage of elderly people in poor health who name pensions for the elderly and retired veterans as their main income source is less than 20 %, while the proportion of the older people who mainly depend on the support of other family members is up to around 70 %. Chinese elderly people enjoy low social security, and more needs to be done to help the elderly in this respect.

### Daily care

Traditionally, an elderly person’s spouse and children are his or her main caregivers, and spouses can take care of the daily life of the elderly who need care. Nevertheless, many elderly people are widowed. According to data from the 2010 population census, 41 million elderly people are widowed in China, accounting for 34 % of all elderly people, including 12.01 million elderly men (accounting for 21 % of all elderly men) and 28.98 million elderly women (accounting for 47 % of all elderly women). In rural areas, 24.50 million elderly people are widowed, accounting for 37 %; 7.67 million elderly men are widowed, accounting for 24 %; 18.63 million elderly women are widowed (49 %) (PCO 2012). For men, the average age of widowhood is 72.6, and after becoming widowed, men may survive 11.2 years; for women, the average age of widowhood is 70.6, and after becoming widowed, women may survive 14.7 years. In rural areas, the average age of widowhood for men is 68.3, and after becoming widowed, rural men may survive 13.0 years; the average age of widowhood for women is 66.3, and after becoming widowed, rural women may survive 17.0 years (Jiang et al. 2015).

In China, traditionally, children are the main caregivers for the elderly. However, traditional elderly care within the family is suffering a huge impact. Firstly, with social and economic development and the implementation of the family planning policy, the number of children has fallen. According to data from the 2010 population census, the average number of surviving children is 2.60, 2.18, 1.94 and 1.82 for 60–64 year-old, 55–59 year-old, 50–54 year-old, and 45–49 year-old women (PCO 2012). However, as social competition becomes even fiercer, along with the accelerating pace of life, the time cost of taking care of an older person for their adult children is increasingly high, and the long-term care could easily result in family conflict and psychological problems (Tang and Lou 2010). Secondly, the family planning policy that has been implemented over the last 35 years has produced about 160 million only-child families, and has also led to numerous families who are bereft of their only child. Eventually (if the mother reaches the age of 90) the risk of losing a male child for a mother is 14.94 %, while that for a female child is 12.21 %. The average age of the mother’s loss of a male child is 57 years old and 51.44 years old for a female child. The time the mother survives after this bereavement is 25 years for a male child, and 30 years for a female child (Jiang et al. 2014). Statistics estimate that at least one to two million families have lost their only child in China. It has been predicted that the number of families bereft of their only child may reach 11.84 million by 2050 (Wang 2013a). Parents who have lost their only child are vulnerable in their physical health, psychological health, economic situation, and social networks. They actively or passively self-exclude themselves from the outside world (Wei et al. 2016).

Due to the decline in the number of children, the traditional pattern of daughters getting married and moving out, as well as the current large-scale influx of rural residents into cities, it is becoming more and more usual for elderly people to live alone. The 2010 census data indicated that among people aged 65 and over, 14.44 million households of elderly people live alone, and 8.12 million of such elderly people are in rural areas; 13.53 million households are elderly couple households, and 6.84 million such households live in rural areas (PCO 2012).

An increasingly serious “empty nest” phenomenon poses a great challenge in terms of the daily care of the elderly and is giving rise to some extreme social problems. For example, on November 24, 2015, in Xialu District of Huangshi City of Hubei Province, an elderly unmarried man was found to have died in his home more than 6 years before he was discovered: only his skeleton was left (Mu 2015).

As China is becoming an aging society, there is a growing demand for care for the elderly, especially for those who are disabled. However, with the decline in family size and rise in rural-to-urban migration of young people, the availability of family members to provide care and support to elderly parents will most likely continue to decrease (Wu et al. 2009). The unprecedented weakening of family caregiving and the relative absence of community and institutional care make care for disabled elderly an important social issue which urgently needs to be solved.

### High suicide rates among the elderly population

Because of changes in family structure and intergenerational relations, the structural authority of the elderly has been undermined, but appropriate protection mechanisms have not been established for those elderly who are in a weak position. With the decline of traditional filial piety and respect for the elderly, the elderly are increasingly being regarded as consumers of resources within the family and in society as a whole (Li et al. 2009). When the elderly lose the capacity to work, caring for them becomes a major difficulty. Especially for the rural elderly, there are few sources of care. Once they cannot take care of themselves, if there are neither children to take care of them, nor a nursing house to go to, they may become desperate and commit suicide.

The elderly commit suicide mainly because of either their hard life or the pain of disease, these two reasons accounting for 60 % of direct causes of death. Another important factor leading to suicide among the elderly relates to their emotional problems. As in the fierce competitive society, middle-aged people are anxious to seek how they can lighten the load in market societies so as to stand out. Undoubtedly, the elderly, who are even more vulnerable, seem to become a burden to their adult children. “I could hardly bear my own burdens, let alone care for the elderly,” some farmers are quite straightforward with their indifference to their elderly parents during the interview. Actually, most of the older people committing suicide did not want to die (Xuan 2014).

What makes this even more shocking is that villagers are indifferent to the suicide of elderly people, and many people think it is a normal phenomenon. Many villagers even think that the suicide of seriously sick or paralyzed elderly people is good for their children (Liu 2014; Yan 2015). Both the survey and case study have shown that since the middle of the 1990s, suicide among rural elderly people has become increasingly problematic. It is predicted that in the next 10–20 years, the suicide rate among rural elderly people will be aggravated (Liu 2014). The suicide rates for older people in rural areas for 2004–2013 are presented in Table 3.

**Table 3:** Suicide rates for older people by age group in rural China (per 100,000)

Years	Age group				
	65–69	70–74	75–79	80–84	85–89
2004	30.24	44.88	59.68	89.53	107.62
2005	39.15	42.89	65.16	82.98	97.50
2006	26.35	36.11	48.02	65.82	74.03
2007	28.45	40.55	57.21	86.73	97.52
2008	20.51	26.93	41.18	51.87	62.48
2009	23.68	35.10	53.00	81.16	104.92
2010	27.02	44.25	68.78	108.13	191.74
2011	25.59	35.23	46.36	78.50	93.46
2012	19.79	26.58	43.47	53.08	71.17
2013	22.06	30.96	43.95	59.18	72.40

*Data source* Annual China Health Statistics Yearbook

Rural vs urban differences in potential years of life lost (PYLL) due to suicide are large. The PYLL due to suicide was approximately twofold higher in rural areas compared with urban areas (Sun and Zhang 2015). In China’s vast rural areas, especially in the economically underdeveloped regions, there are many family tragedies where the elderly commit suicide, which indicates the poor situation of the aged in rural areas. It critical to solve this problem and help the country deal with its aging population (Ge 2016).

## Insufficient social security system

### Pension

According to data from the 2010 population census, 51 % of urban elderly people rely mainly on pensions for the elderly and retired veterans, 35 % of urban elderly people rely mainly on the support of other family members, and 8 % name their labor income as their main source of support (PCO 2012). 51 % of urban elderly people rely mainly on pensions for the elderly and retired veterans because urban areas implement the basic endowment insurance system for urban workers. In 2014, the average monthly pension for the elderly and retired veterans (according to the basic endowment insurance for workers) was 2,061 yuan (318USD per month). By the end of 2014, the number of people buying the basic endowment insurance for urban workers was only 341.24 million (Caijing Net 2015).

However, what most people buy is a relatively low security level of basic endowment insurance for urban and rural residents. Before this, there was no endowment insurance system for the urban unemployed population and the rural population. In September 2009, China launched the pilot project for a new rural social endowment insurance; in July 2011, China launched the pilot project for an urban resident endowment insurance. In 2014, these two pilot projects were combined to create an integrated basic endowment insurance system for urban and rural residents. By the end of 2014, the number of people participating in basic endowment insurance for urban and rural residents had reached 501.07 million. But the security level of such social insurance is relatively low. In 2013, the monthly per capita pension provided by the basic endowment insurance for urban and rural residents was only 82 yuan; in 2014, it was 90 yuan (14 USD per month) (Caijing Net 2015), which is far from enough to pay for someone’s daily needs. This basic endowment insurance is basically a financial transfer payment, that is, it is not an insurance system, but mainly a welfare system (Li 2014).

In view of this situation, Hu Xiaoyi, the vice minister of Ministry of Human Resources and Social Security of the People’s Republic of China stated that a low level of social security is an objective fact; for the majority of farmers: the main source of income still comes from land income and family income, as well as the wages of family members through migrant work, while the basic pension is just a supplementary income (Li 2014).

With the effects of population aging gradually becoming noticeable, the growth in the number of retirees insured is higher than that of the number of contributors. In some areas, the dependency ratio is relatively high. As a result, the social security fund expenditure increases rapidly. In 2014, income failed to cover the expenditure on pensions in 22 out of 30 mainland provinces. The gap has to be filled with a financial subsidy from all levels of government. In fact, in 13 years from 2002 to 2014, the financial system at all levels has provided a total subsidy for pensions amounting to 2.0748 trillion yuan. In 2002, the subsidy amount was 40.82 billion yuan; in 2011, it reached 227.2 billion yuan; in 2013, it rose to 301.9 billion yuan; in 2015, the budgeted subsidy amount was 367.12 billion yuan (Guo 2015).

The Chinese government has already made attempts to solve the plight of the elderly. According to Decision of the Central Committee of the Communist Party of China on Some Major Issues Concerning Comprehensively Deepening the Reform passed at the Third Plenary Session of the 18th Central Committee of the Communist Party of China held in November 2013, we should uphold the principle of balance based on actuarial mathematics; the financial department hopes the future endowment insurance reform will reduce the dependence on finance and strike a self-regulating balance. Lou Jiwei, the minister responsible for the Ministry of Human Resources and Social Security of the People’s Republic of China, interpreted the proposal for the thirteenth Five-Year Plan, and stated that the next step for extending social security, especially the social insurance system reform, should be to uphold the principle of balance based on actuarial mathematics, to promote a social insurance fund to strike a self-regulating balance, and organize a long-term, stable operation (Guo 2015). However, there is no specific feasible proposal on how to achieve such a balance.

### Health expenses for the elderly

Per capita health spending of the elderly aged 65 and over is twice to eight times more than that of people below 65 years old. The proportion of the older people using outpatient treatment services has increased from 27 to 49 % during the past decade, and the demand for inpatient treatment services and health care also shows upward trends. Total healthcare expenditure on the elderly as a percentage of GDP increased from 2.1 % in 1993 to 3.4 % in 2013. Health-related bills are the major driver pushing up the elderly’s consumption. Due to the changing population age structure, the demand for healthcare services increased by 15.2 % in 2015 compared with a decade ago, and the number of chronic illness cases jumped by 31.1 % (Li 2016).

The Chinese government has announced three different types of health insurance programs, which include Urban Employee Basic Medical Insurance (UEBMI), Urban Resident Basic Medical Insurance (URBMI), and the New Cooperative Medical Scheme (NCMS). The UEBMI, which all urban employees are required to participate in, started in 1998. The NCMS, initiated in the early 2000s, is for voluntary rural participants. Urban residents who are not eligible for UEBMI should participate in URBMI (Cai and Du 2015). On March 5th, 2015, Premier Li Keqiang in his report on the work of the government stated that by 2014 universal health coverage in China has exceeded 95 % (Ma 2015). Medical insurance is becoming an important channel to pay the health care bills of the elderly. However, considering the practical effects of the insurance scheme, there is no evidence that the NCMS has reduced their out-of-pocket spending (Cheng et al. 2015). People with UEBMI and URBMI are more likely to use outpatient services and people with UEBMI have less out-of-pocket payments in Zhejiang, but there is no evidence that NCMS increases utilization of outpatient and inpatient services, nor does it decrease the out-of-pocket payments in Zhejiang and Gansu provinces (Li and Zhang 2013). The lack of improvement in health care services could be attributed to the following reasons: first, patients may be prevented from using insurance for some treatments, as the reimbursement process can be lengthy and complicated under the current healthcare system in China. Second, even though insurance utilization can reduce out-of-pocket medical expenditure, the amount paid out of pocket is still high for the insured (Wang 2013b).

The UEBMI’s financing and reimbursement are at relatively high level, while those of the URBMI and the NCMS are rather low. Also, some retirees feel the reimbursements they receive are inadequate because of their high medical expenses (Wang 2011). The present NCMS policies, on the one hand, have reduced the situation that means that it is often difficult and expensive for the rural elderly to access medical treatments; on the other hand, problems still exist, such as the low reimbursement rates, the fact that only a few items of medical expenditure are eligible for reimbursement and there are limited medical resource in counties and townships. The very problem most widely reported by the rural elderly is still that it is hard to get proper medical treatment in the local community, while going to cities, the medical costs cannot be covered under the insurance (Wang 2013b).

An aging population significantly changes the age structure of the insured. With the increasing number of older people, healthcare expenditure for the elderly grows rapidly; in the meantime, relatively few people will pay health insurance due to population aging, making it more difficult to pay for health insurance. As Cai and Du (2015) has mentioned, no matter what types of pension schemes are applied, an aging society has to have faster growth in labor productivity than its speed of aging and more resources are needed to support the livelihood of the elderly.

### Long term care costs for the elderly

By the end of 2014, China had nearly 40 million elderly people aged 60 and above who were completely disabled or semi-disabled. This means that there is a growing demand for medical and nursing care as well as rehabilitation services, which poses huge challenges for China’s pension and health care systems (Li 2016). Due to the one-child policy, rural/urban migration and other societal changes, the family-dependent long-term care (LTC) of the past will no longer suffice (Glass et al. 2013). Family caregiving will need to be supplemented on a large scale by more formal LTC (Wu et al. 2009).

The Chinese government places great emphasis on the establishment of the long-term care system for the elderly. As clearly stated in the Law on Protecting the Rights and Benefits of Older Persons of the People’s Republic of China, which was revised in 2012, the State will gradually promote the development of long-term care to meet the increasing demand from the elderly. It was further pointed out in the fifth plenary session of the 18th Communist Party of China Central Committee held in October 2015 that China will explore establishing a long-term care insurance system (Han 2015). However, though formal LTC systems are emerging, they are still at the preliminary stages of development (Wu et al. 2009). Many Chinese seniors who are interested are still unable to use institutional LTC due to the high cost, concerns about service quality, and moral beliefs that oppose it (Glass et al. 2013). Meanwhile, many rural elders are left to manage their LTC needs themselves or are dependent solely on family support due to limited financial resources (Zhai and Ren 2007). Furthermore, although China has expanded its social security systems through implementing its current low-level basic old-age insurance system and health security system with wide population coverage, the country still fails to cover the cost of long-term care for the elderly in the systems. Moreover, commercial insurance is much less involved in this field. The old people and their adult children tend to feel more pressure in the face of the high cost of long-term elderly care as it is paid either by older people’s accumulated savings or by their children’s financial support.

## Rapid aging in future

For some time in the foreseeable future, China’s population will age more quickly. The number and percentage of China’s elderly population according to the United Nations’ prediction are as displayed in Table 4.

**Table 4:** China’s population aging (Unit: Million,  %)

Years	People aged 65 and over		People aged 80 and over		Elderly dependency ratio
	Number	Percentage	Number	Percentage	
2015	131.43	9.55	22.36	1.62	13.05
2020	169.61	12.09	26.92	1.92	17.08
2025	200.60	14.18	31.52	2.23	20.38
2030	243.17	17.18	41.41	2.93	25.26
2035	299.29	21.25	59.97	4.26	32.66
2040	342.92	24.59	72.70	5.21	39.60
2045	357.83	26.03	92.27	6.71	42.98
2050	371.39	27.55	120.57	8.94	46.74

*Data source* United Nations (2015)

According to the United Nations data and prediction results (United Nations 2015), for the percentage of people aged 65 and over within the total population to increase from 10 to 20 % it will take: 18 years in China (2016–2034); 22 years in Japan (1984–2006); 57 years in Germany (1951–2008); 68 years in Sweden (1947–2015); 56 years in the United States of America (1972–2028). All these developed countries took or will take a longer time for the proportion of older people to double from 10 percent.

According to a report published in 2013, the number of older people with functional disabilities exceeded 37.50 million, those with chronic diseases more than 100 million, older people in an “empty nest” exceeded 100 million, 23 million older people lived under the poverty line, and 50 million older people were left behind in rural areas (Wu and Dang 2013). Furthermore, those numbers are expected to increase in the years to come.

## Conclusions

In the Decision of the Central Committee of the Communist Party of China on Some Major Issues Concerning Comprehensively Deepening the Reform, passed at the Third Plenary Session of the 18th Central Committee of the Communist Party of China held in November 2013, it was stated that China would respond actively to the aging of the population, quicken steps to establish a social endowment service system, and develop the service industry for the elderly. In recent years, the Chinese government has energetically promoted new social insurance projects, to provide better security for the elderly. Public medical insurance, in particular, which is important for the elderly, has almost reached full coverage, and the endowment insurance program is also being popularized throughout the whole country.

Nevertheless, as can be seen from the discussion above, the security level of these projects and programs is relatively low and they are far from paying all medical expenses or living expenditure after retirement for most rural elderly people. There will still be problems and challenges for China’s elderly people in the foreseeable future: low security and income level and a weak ability to resist risks; significant growth in demands for care giving and a shortage of care-giving service resources; many health risks and a heavy medical burden; increasing problems adapting living arrangements inclined to independent living and the phenomenon of the “empty nest”. For a long time, the spouse and children will be the main caregivers for elderly people. With the advent of small families and the decline in the number of children in a family, the trend for family members to take complete care of the elderly will be difficult to sustain. Because of the dramatic decline and low level in fertility during the past several decades, China has benefited notably in terms of economic and social development because of an enormous demographic dividend. However, with the closure of the opportunity window of the population, as well as increasing life expectancy, it will be very difficult for China to afford its rapidly aging population.

## References

[CR1] Caijing Net (2015) Ministry of human resources and social security: per capita monthly old age pension 90 Yuan. June 30 2015. Accessed Feb 20 2016. http://economy.caijing.com.cn/20150630/3915534.shtml

[CR2] Cai F, Du Y (2015). The social protection system in ageing China. Asian Econ Policy Rev.

[CR3] CHARLS Research Team (2013) Challenges of population aging in China: evidence from the national baseline survey of the China Health and Retirement Longitudinal Study (CHARLS). Accessed Feb 20 2016. http://cmrc.nsd.pku.edu.cn/cn/userfiles/Other/2013-06/2013060317093161771377.doc

[CR4] Cheng L (2015). The impact of health insurance on health outcomes and spending of the elderly: evidence from China’s new cooperative medical scheme. Health Econ.

[CR5] Du P (2013). An analysis on the health status of the older persons in China. Popul Econ.

[CR6] Ge J (2016). The first foundation devoted to rural empty-nest older people. Soc Public Welf.

[CR7] Glass AP, Gao Y, Luo J (2013). China: facing a long-term care challenge on an unprecedented scale. Global Public Health.

[CR8] Gu D, Sautter J, Pipkin R, Zeng Y (2010). Sociodemographic and health correlates of sleep quality and duration among very old Chinese. Sleep.

[CR9] Guo J (2015) Avenue less than expenditure in old age pension in 22 provinces. China Business New. November 23 2015. Accessed Feb 20 2016. http://www.yicai.com/news/2015/11/4715255.html

[CR10] Han B (2015) Explore to establish the long-term care system for older people. ChinaEconomic Net. December 17 2015. http://www.ce.cn/xwzx/gnsz/gdxw/201512/17/t20151217_7628762.shtml

[CR11] Jiang Q, Li Y, Sánchez Barricarte JJ (2014). The risk of mothers losing an only child in China. J Biosoc Sci.

[CR12] Jiang Q, Li X, Sánchez Barricarte JJ (2015). Elderly widowhood in China. Asian Popul Stud.

[CR13] Li T (2014) Monthly old age pension only 81 Yuan, less than one seventh of rural basic living allowances. Economic Information Daily, April 14 2014, P. A03. Accessed Feb 20 2016. http://dz.jjckb.cn/www/pages/webpage2009/html/2014-04/14/content_88955.htm?div=-1

[CR14] Li H (2016) Getting older before getting rich: people aged 60 and above exceed 212 million, almost 40 million with functional disability. People’s Daily, Feb 29 2016. http://paper.people.com.cn/rmrb/html/2016-01/29/nbs.D110000renmrb_04.htm

[CR15] Li X, Zhang W (2013). The impacts of health insurance on health care utilization among the older people in China. Soc Sci Med.

[CR16] Li X, Xiao Z, Xiao S (2009). Suicide among the elderly in mainland China. Psychogeriatrics.

[CR17] Liu Y (2014). Peasant’s suicide in rural China.

[CR18] Ma X (2015) Premier Li Keqiang: the coverage rate of medical care system exceeds 95 percent. People.cn. March 5 2015. http://health.people.com.cn/n/2015/0305/c14739-26642218.html

[CR19] Mu G (2015) Lonely and bitter older people in empty nest. Xinmin Evening News. December 6 2015. P. C4. Accessed Feb 20 2016. http://xmwb.xinmin.cn/xmwb/html/2015-12/06/content_32_2.htm

[CR20] Population Census Office under the State Council (PCO) (2002). Tabulation on the 2000 population census of the People’s Republic of China.

[CR21] Population Census Office under the State Council (PCO) (2012). Tabulation on the 2010 population census of the People’s Republic of China.

[CR22] Sun L, Zhang J (2015). Potential years of life lost due to suicide in China, 2006–2010. Public health.

[CR23] Tang Y, Lou W (2010). The effect of and challenge to family and community of older people’s long-term care need-the case in Shenzhen. Lanzhou J.

[CR24] United Nations (2015) Probabilistic Population Projections based on the World Population Prospects: The 2015 Revision. Population Division, DESA. http://esa.un.org/unpd/ppp/

[CR25] Wang Q (2011) The coverage of medical care system to be extended. Oct 13 2011. http://www.chinadaily.com.cn/hqgj/jryw/2011-10-13/content_4054948.html

[CR26] Wang G (2013). “Only-child-death” family and its developing trends under the current family planning policy. Chin J Popul Sci.

[CR27] Wang M (2013b) The solution for rural old age support. China Youth’s Daily, P.03. Nov 13 2013. http://zqb.cyol.com/html/2013-11/13/nw.D110000zgqnb_20131113_1-03.htm

[CR29] Wei Y, Jiang Q, Basten S (2016). The wellbeing of bereaved parents in an only-child society. Death Stud.

[CR30] Wu Y, Dang JW (2013). China report of the development on the aging cause.

[CR31] Wu B, Mao Z, Zhong R (2009). Long-term care arrangements in rural China: review of recent developments. J Am Med Dir Assoc.

[CR32] Xuan J (2014) A sociological study of rural older people’s suicide. China Youth Daily. July 30 2014. http://zqb.cyol.com/html/2014-07/30/nw.D110000zgqnb_20140730_3-09.htm

[CR33] Yan H (2015). Report from Yabian: the fracture record of rural China.

[CR34] Zhai X, Ren Z (2007). Perceptions of long-term care, autonomy, and dignity, by residents, family and caregivers: the Beijing experience. J Med Philos.

[CR35] Zhao Y, Smith JP, Strauss J (2014). Can China age healthily?. Lancet.

